# Comparative Study of Blood-Based Biomarkers, α2,3-Sialic Acid PSA and PHI, for High-Risk Prostate Cancer Detection

**DOI:** 10.3390/ijms18040845

**Published:** 2017-04-17

**Authors:** Montserrat Ferrer-Batallé, Esther Llop, Manel Ramírez, Rosa Núria Aleixandre, Marc Saez, Josep Comet, Rafael de Llorens, Rosa Peracaula

**Affiliations:** 1 Biochemistry and Molecular Biology Unit, Department of Biology, University of Girona, 17003 Girona, Spain; montserrat.ferrer@udg.edu (M.F.-B.); esther.llop@udg.edu (E.L.); 2 Girona Biomedical Research Institute (IDIBGI), 17190 Salt (Girona), Spain; jramirez.girona.ics@gencat.cat (M.R.); rnaleixandre@infonegocio.com (R.N.A.); 28547jcb@comb.cat (J.C.); 3 Catalan Health Institute, University Hospital of Girona Dr. Josep Trueta, 17007 Girona, Spain; 4 Research Group on Statistics, Econometrics and Health (GRECS), University of Girona, 17003 Girona, Spain; marc.saez@udg.edu; 5 CIBER of Epidemiology and Public Health (CIBERESP), 28029 Madrid, Spain

**Keywords:** diagnosis, glycosylation, prostate cancer, prostate specific antigen, proPSA, PHI, α2,3-sialic acid

## Abstract

Prostate Specific Antigen (PSA) is the most commonly used serum marker for prostate cancer (PCa), although it is not specific and sensitive enough to allow the differential diagnosis of the more aggressive tumors. For that, new diagnostic methods are being developed, such as PCA-3, PSA isoforms that have resulted in the 4K score or the Prostate Health Index (PHI), and PSA glycoforms. In the present study, we have compared the PHI with our recently developed PSA glycoform assay, based on the determination of the α2,3-sialic acid percentage of serum PSA (% α2,3-SA), in a cohort of 79 patients, which include 50 PCa of different grades and 29 benign prostate hyperplasia (BPH) patients. The % α2,3-SA could distinguish high-risk PCa patients from the rest of patients better than the PHI (area under the curve (AUC) of 0.971 vs. 0.840), although the PHI correlated better with the Gleason score than the % α2,3-SA. The combination of both markers increased the AUC up to 0.985 resulting in 100% sensitivity and 94.7% specificity to differentiate high-risk PCa from the other low and intermediate-risk PCa and BPH patients. These results suggest that both serum markers complement each other and offer an improved diagnostic tool to identify high-risk PCa, which is an important requirement for guiding treatment decisions.

## 1. Introduction

Prostate cancer (PCa) is an important problem in public health and a major disease that affects men’s health worldwide. It was the most commonly diagnosed male neoplasia in western countries and Japan last year. It is expected that around one of each six men will be diagnosed with PCa during his life. In addition, as the number of older people increase, the incidence of the disease will raise dramatically in the coming decades [1].

The serum marker Prostate Specific Antigen (PSA), adopted in the early 1990’s, has been the widely used and preferred assay for prostate diseases, including PCa, with important levels of success, and represents the gold standard marker as an essential tool for urologists [1,2]. In addition, since PCa has a long natural history, the PSA assay predicts a prostate pathology decades before a confirmatory diagnostic [3]. This means that a majority of men diagnosed with PCa could be detected at early stages and with localized prostate cancer. Epidemiologic studies indicate an important and continuous decrease in prostate cancer mortality since the application of the PSA screening test [4]. 

However, the PSA test presents some limitations. It is organ specific but not cancer specific [5]. Serum PSA levels could also be elevated in benign prostate hyperplasia (BPH), prostatitis, and prostate manipulations (as DRE and bicycling), and cannot discriminate between aggressive and non-aggressive cancers. PSA assays present a high rate of false positives that leads to over-diagnosis, unnecessary biopsies, and over-treatments [6]. Actually only 25% of men biopsied after an elevated PSA level have PCa, and many of these cancers are slow growing, with no impact in the patient’s life [7].

New non-invasive biomarkers with greater sensitivity and specificity that are capable of distinguishing aggressive tumors from indolent ones are required [5]. To improve the specificity of the PSA as a biomarker, different strategies using several PSA isoforms (ratio free PSA/total PSA, PSA density and velocity, proPSA forms, 4K score, and Prostate Health Index (PHI)) have been developed [7], recently including, PSA glycoforms [8,9,10].

Regarding proPSA forms, these were first identified in the serum of patients with prostate cancer in 1997 [11]. ProPSAs were preferentially elevated in the peripheral zone of prostatic tissue containing cancer, whilst remaining largely undetectable in the transitional zone of the prostate [12]. These proPSA forms comprised the complete sequence of inactive zymogen [−7]proPSA, and also shorter forms as [−5] [−4] and [−2]proPSA. [−2]proPSA was present in the sera of prostate cancer patients and it was a more specific serum marker that could improve a PSA assay. [−7] and [−5]proPSA did not give adequate results as biomarkers [13]. The interesting positive results of the [−2]proPSA detection moved Beckman & Coulter Inc. (Brea, CA, USA), in partnership with the NCI Early Detection Research Network, to develop a mathematical algorithm with [−2]proPSA, tPSA, and fPSA serum levels, the so called Prostate Health Index: PHI = ([−2]proPSA/fPSA) × $\sqrt{\mathrm{tPSA}}$ [14]. PHI received the FDA approval in 2012 [7]. Several works, including numerous international multicenter studies, have indicated that PHI score outperforms its individual components for the prediction of overall and high-grade prostate cancer [6,15,16,17]. PHI score has a high diagnostic accuracy rate and may be useful as a tumor marker in predicting patients harboring more aggressive disease. PHI also predicts the likelihood of progression during active surveillance. PHI score has been reported to correlate with PSA serum levels and Gleason scores. Nowadays, PHI has regulatory approval in more than 50 countries worldwide and is now being incorporated into prostate cancer guidelines for early prostate cancer detection and risk stratification [18]. However, others studies do not completely agree with these results and indicated that when the goal is to detect at least 95% of the aggressive tumors, PHI does not seem to be much more effective than the %fPSA and the PSA density [19]. 

To address the problem of the discovery of new non-invasive PCa markers that can predict PCa aggressiveness, several authors have determined the glycosylation pattern of PSA from healthy donors, PCa cancer cell lines, and PCa serum patients, and have shown specific changes in the PSA core fucosylation and sialylation levels in PCa patients [8,10,20,21,22,23,24,25,26,27]. In this regard, we have developed a methodology to quantify the ratio of core fucosylation of serum PSA and the percentage of α2,3-sialic acid of serum PSA and have shown a decrease in the content of core fucose and an increase in α2,3-sialic acid of PSA *N*-glycans in patients with high-risk PCa [9]. In particular, the percentage of α2,3-sialic acid of PSA was increased in the high-risk PCa patients compared with low or intermediate-risk PCa and BPH patients and gave an AUC of 0.971, with 85.7% sensitivity and 95.3% specificity. Interestingly, the percentage of α2,3-sialic acid of PSA also correlated with the Gleason score of PCa patients.

With the aim of searching for new serum markers that could assist in the identification of the aggressive prostate cancers, the present study compared the potential of PHI and the percentage of α2,3-sialic of PSA, alone and in combination, to identify high risk PCa cancer in a cohort of 79 patients’ serum samples.

## 2. Results

### 2.1. Clinical and Pathological Characteristics of the Patients 

A cohort of 79 serum samples containing 29 BPH and 50 PCa samples was used for the study of the two blood-based biomarkers, PHI and the percentage of α2,3-sialic of PSA. PCa staging was determined according to the International Union Against Cancer (IUAC) and patients were classified in high-risk (*N* = 22), intermediate-risk (*N* = 21) and low-risk (*N* = 7). Clinical data of the subjects included in this study are summarized in Table 1.

The seven low-risk PCa patients had tPSA levels below 10 ng/mL and Gleason scores ≤6. The 21 intermediate-risk PCa group comprised five patients with a Gleason score of six and clinical stage >pT2a; 15 patients with a Gleason score of seven and one subject presenting a focal Gleason score of eight. Their tPSA levels were between 3.73 and 12.42 ng/mL. The 22 high-risk PCa included 18 with a Gleason score ≥8, two with a Gleason score of seven and metastasis, and two other subjects with an undetermined Gleason score who also presented metastasis. Data corresponding to the age and total and free PSA values of all groups of patients are shown in Table 1.

Evaluation of the clinical outcome of the PCa patients showed a PCa recurrence one year after treatment of 0%, 4.8%, and 59% in the low, intermediate, and high-risk PCa groups respectively. Data of the five-year relapse-free survival was reported for all patients in the low-risk group being 100%. However, this information was not available for all patients in the other two groups. The five-year relapse-free survival was 95% for the intermediate-risk group corresponding to 20 out of the 21 patients, and it was 40% in the high-risk group corresponding to 15 out of the 22 patients.

### 2.2. Analysis of α2,3-Sialic Acid PSA in Serum Samples

For the analysis of percentage of α2,3-sialic acid PSA, 0.75 mL of each serum were required. First, the serum samples were treated with ethanolamine, in order to release PSA from its complex with α1-antichymotrypsin. Then, total PSA from the serum samples was immunoprecipitated and loaded into a SNA lectin column. This lectin chromatography, which binds to α2,6-sialylated glycoconjugates, allows for the separation of α2,3-sialylated from α2,6-sialylated PSA glycoforms [9]. After the lectin chromatography, free PSA in the unbound (α2,3-sialylated PSA) and bound fractions (α2,6-sialylated PSA) was measured, and from these data the percentage of fPSA in both fractions was calculated. The percentage of the unbound fraction corresponded to the percentage of α2,3-sialic acid PSA.

The potential of the percentage of α2,3-sialic acid PSA as a blood biomarker for aggressive PCa was assessed in the cohort of sera (29 BPH, seven low-risk, 21 intermediate-risk and 22 high-risk PCa). Three different PCa serum samples, containing different values of tPSA (12.87, 23.08, and 40.61 ng/mL) were repeatedly analyzed in the different batches of samples in order to calculate the inter-assay variation of the method that was lower than 12%.

**Figure 1:**
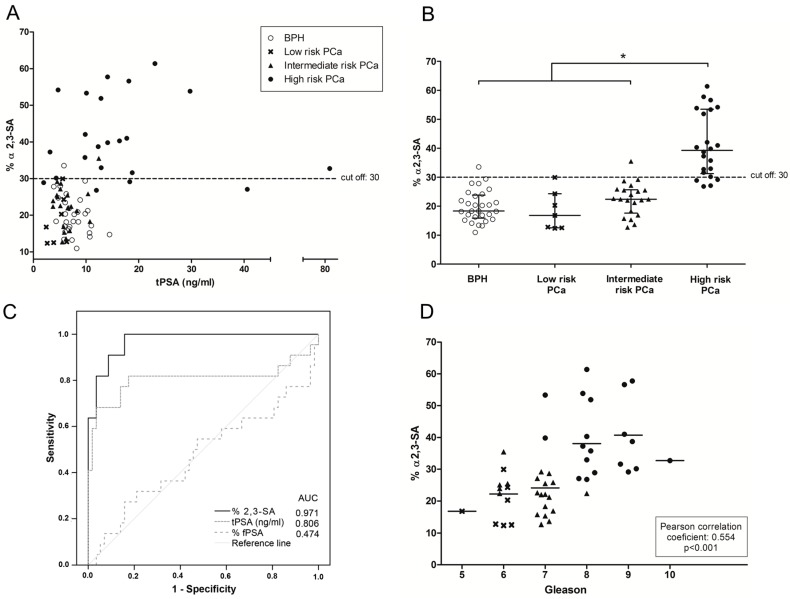
α2,3-SA percentage of Prostate Specific Antigen (PSA) (% α2,3-SA) of the cohort of 79 serum samples. Benign Prostate Hyperplasia (BPH) samples are represented with an open circle (ο), low risk PCa with a cross (**×**), intermediate risk PCa with a filled triangle (▲) and high risk PCa with a filled circle (●). (**A**) Representation of % α2,3-SA against tPSA serum levels; dotted line (---) shows the cutoff value for discriminating high risk PCa samples from the other three groups; (**B**) Representation of % α2,3-SA against the pathology. The center line indicates the median, and the top and bottom lines, the 75th and 25th percentiles, respectively; (**C**) Representation of the Receiver operating characteristic (ROC) curves for % α2,3-SA, tPSA, and %fPSA; (**D**) Correlation plot of % α2,3-SA from the PCa serum samples with their Gleason score. The mean of % α2,3-SA of each Gleason score is shown with a horizontal line (-).

The plot of the percentage of α2,3-sialylated PSA is represented against the concentration of the total PSA of each sample (Figure 1A) and in the four groups (Figure 1B). A significant increase of percentage of α2,3-sialylated PSA in the group of high-risk PCa patients (26.8–61.4%) compared with the other three groups, intermediate-risk PCa (12.7–35.5%; *p* < 0.001), low-risk PCa (12.3–29.9%; *p* = 0.006), and BPH (10.9–33.5%; *p* < 0.001) was shown. However, no significant differences were found between BPH and low and intermediate-risk PCa patients. The correlation of α2,3-sialylated PSA values of the samples with their corresponding tPSA levels was tested and resulted to be non-significant in any of the BPH and PCa groups. Both parameters were then independent, indicating that a high or a low percentage of α2,3-sialylated PSA could be found in sera with either low or high tPSA levels in any group of patients (Figure 1A).

In order to compare the performance of PSA α2,3-sialic acid percentage with that of tPSA and the %fPSA values, the Receiver operating characteristic (ROC) curves of these three parameters were compared (Figure 1C). The ROC assay showed that % α2,3-sialic acid had the highest performance and could separate high-risk PCa patients from BPH, low, or intermediate-risk prostate cancers with 81.8% sensitivity and 96.5% specificity with a cutoff of 30%, resulting in an AUC of 0.97. In addition, this biomarker, which is based on the detection of specific PSA glycoforms, significantly correlated with the Gleason score of the tumor (correlation coefficient 0.554, *p* < 0.001) (Figure 1D), which highlights its potential as a marker for aggressive PCa.

### 2.3. Prostate Health Index (PHI) Score Analysis of Serum Samples

For this analysis, patients’ sera were analyzed for total PSA (tPSA), free PSA (fPSA), and [−2]proPSA. Then the Prostate Health Index (PHI) score was calculated [PHI = ([−2]proPSA /fPSA) × $\sqrt{\mathrm{tPSA}}$]. This methodology was used to analyze the cohort of serum samples tested previously for α2,3-sialic acid percentage of PSA.

**Figure 2:**
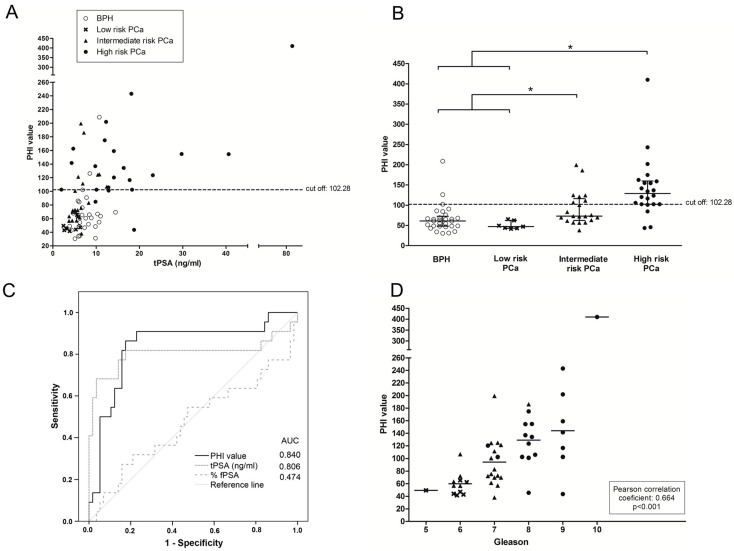
Prostate Health Index (PHI) values of the cohort of 79 serum samples. BPH samples are represented with an open circle (ο), low risk PCa with a cross (**×**), intermediate risk PCa with a filled triangle (▲) and high risk PCa with a filled circle (●). (**A**) Representation of PHI value against tPSA serum levels; dotted line (---) shows the cutoff value for discriminating high risk PCa samples from the other three groups; (**B**) Representation of PHI value against the pathology. The center line indicates the median, and the top and bottom lines, the 75th and 25th percentiles, respectively; (**C**) Representation of the ROC curves for the PHI value, tPSA and %fPSA (**D**) Correlation plot of PHI value of the PCa samples with their Gleason score. The mean PHI value of each Gleason score is shown with a horizontal line (-).

The plot of the PHI score is shown against the concentration of total serum PSA of each sample (Figure 2A) and in the four groups (Figure 2B). There was a significant increase of PHI score in the group of high-risk PCa patients compared with the other two groups, low-risk PCa (*p* = 0.006) and BPH (*p* < 0.001). The intermediate-risk PCa group showed also a significant increase of PHI compared with low-risk PCa (*p* = 0.006) and BPH (*p* = 0.022). No significant differences were found between high-risk PCa patients and intermediate-risk PCa neither between BPH and low-risk PCa patients. 

PHI values correlated with the tPSA levels of the sample in the high-risk PCa group (correlation coefficient 0.758, *p* < 0.001), while there was no correlation for the other individual groups.

ROC analysis of the PHI score gave an AUC of 0.840 to discriminate high-risk PCa patients from the other groups, BPH and low- and intermediate-risk PCa. With a PHI cutoff of 102.28, the sensitivity was 81.8% and the specificity was 84.2%. The performance of the PHI score was higher than that of tPSA and %fPSA (Figure 2C). PHI score values showed a significant correlation with the Gleason score of the prostate tumor tissues (correlation coefficient of 0.664; *p* < 0.001) (Figure 2D).

Since PHI values of the high risk group were dependent on tPSA values, a subcohort of patients with tPSA levels lower than 13 ng/mL (*N* = 67, 28 BPH, seven low-risk, 21 intermediate-risk and 11 high-risk PCa) was evaluated. This subcohort reduced basically the number of high-risk PCa patients, which had high levels of tPSA. In this subcohort, there was no correlation of PHI values and tPSA levels within the high-risk group. The AUC of PHI in this subcohort for identifying high-risk PCa was 0.81, slightly lower than when analyzing the whole cohort.

When PHI was assayed to discriminate PCa from BPH, the AUC was of 0.735, sensitivity of 84% and specificity 45%, with a cutoff of 55.7. The diagnostic performance of PHI was higher than tPSA (AUC of 0.506) and %fPSA (AUC of 0.632), in agreement with bibliographic studies. In the subcohort of patients with tPSA levels lower than 13 ng/mL (*N* = 67, 28 BPH, seven low-risk, 21 intermediate-risk and 11 high-risk PCa), PHI performance for PCa diagnosing (AUC of 0.694) was still higher than tPSA (AUC of 0.382) and %fPSA (AUC of 0.630). 

### 2.4. Combinatorial Analysis of PHI and α2,3-Sialic Acid PSA

**Figure 3:**
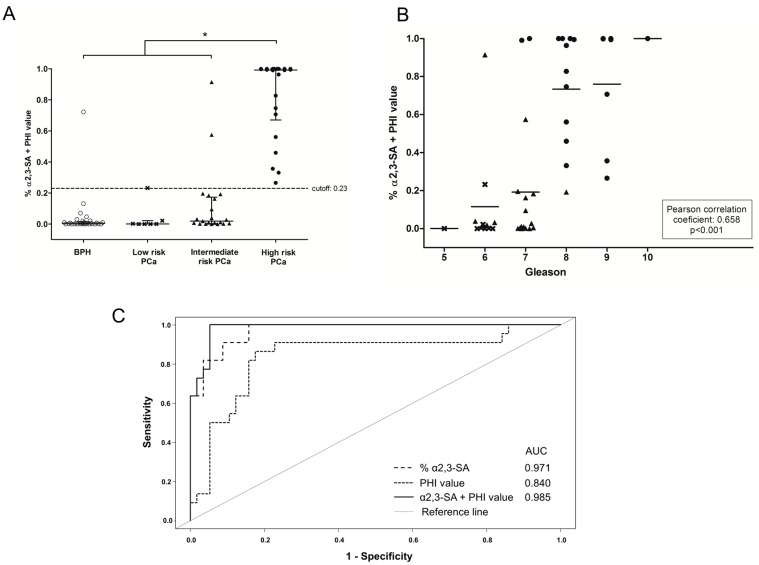
% α2,3-SA and PHI combination of the cohort of 79 serum samples. BPH samples are represented with an open circle (ο), low risk PCa with a cross (**×**), intermediate risk PCa with a filled triangle (▲) and high risk PCa with a filled circle (●). (**A**) Representation of % α2,3-SA and PHI combination values against the pathology. The center line indicates the median, and the top and bottom lines, the 75th and 25th percentiles, respectively; dotted line (---) shows the cutoff value for discriminating high risk PCa samples from the other three groups; (**B**) Correlation plot of % α2,3-SA and PHI combination of the PCa samples with their Gleason score. The mean % α2,3-SA and PHI combination value of each Gleason score is shown with a horizontal line (-); (**C**) ROC curves for the diagnosis of high-risk PCa versus low- and intermediate-risk PCa and BPH. Diagnostic performance of % α2,3-SA and PHI combination (solid line) compared with PHI (dotted line) and % α2,3-SA (dashed line).

In order to assess the performance of the combination of PHI and α2,3-sialic acid PSA, the R statistic package was used. The combination of both biomarkers showed a high performance to differentiate the high-risk PCa group from the other groups with an AUC of 0.985, much higher than PHI alone (Figure 3A,C). The combination of PHI and α2,3-sialic acid PSA also correlated with the Gleason score of the PCa patients and interestingly the two high-risk PCa patients with GS = 7 were classified correctly and were differentiated from 14 out of 15 patients of GS = 7 of the intermediate-risk PCa group (Figure 3B).

With the aim of implementing the combination of PHI and % α2,3-SA in clinics, an algorithm that includes both variables was developed. This consisted of a generalized lineal model (GLM) with a binomial response. After the introduction of PHI and α2,3-sialic acid percentage values, the GLM allowed to classify the patients as high-risk PCa with 100% sensitivity and 94.7% specificity. The cutoff for PHI score was 65.4 and for α2,3-sialic acid percentage of PSA was 29.94%. The model calculates the probability of a patient to be diagnosed as high-risk PCa or not (either low and intermediate-risk PCa or BPH). For a probability equal to, or higher than 23.2% (that corresponds to the point with maximum sensitivity and specificity) the patient will be classified as high-risk PCa with a sensitivity of 100% and a specificity of 94.7%. For a probability lower than 23.2% the patient will be classified either as a low- or intermediate-risk PCa, or a BPH. The probability for each patient is calculated with the following function using the patient values of PHI and α2,3-sialic acid percentage of PSA (% α2,3-SA), where β_0_, β_1_ and β_2_ are parameters estimated by the model:

$$\mathrm{Prob}\left(\mathrm{High}-\mathrm{riskPCa}\right)=\frac{e^{\left(\beta_{0}+\beta_{1}\mathrm{PHI}+\beta_{2}\%\alpha 2,3-\mathrm{SA}\right)}}{1+e^{\left(\beta_{0}+\beta_{1}\mathrm{PHI}+\beta_{2}\%\alpha 2,3-\mathrm{SA}\right)}}.$$

## 3. Discussion

New generation of tumor markers for PCa diagnosis should be able to discriminate between patients with aggressive tumors and those without cancer or low aggressive tumors. Thus, the skills required for the new generation of markers of PCa are high sensitivity and specificity for aggressive tumors. This way, an unnecessary biopsy in men who do not have an aggressive or asymptomatic PCa could be avoided [19,28]. Early diagnosis of PCa frequently, involves the over-detection of non-aggressive tumors.

In the next future, PCa diagnosis and prognosis will probably depend on panels of biomarkers that will allow a more accurate prediction of PCa presence, stage and aggressiveness, so they will be key factors in a clinician making decisions. These markers could include serum non-invasive markers, as well as imaging markers, such as multi-parametric prostate magnetic resonance (mpMRI), which has also been proposed as a means to avoid the incidental detection of low-grade cancers [29,30,31].

PHI is a simple and affordable blood test that could be used as part of a multivariable approach to screening. In this sense, PHI has shown good performance for PCa diagnosis [16]. Our results are in agreement with the reported data and have shown that PHI identifies PCa from BPH with an AUC of 0.735 with higher performance than tPSA (AUC = 0.506) and %fPSA (AUC = 0.632). Since PHI has been recommended for PSA levels between 4–10 ng/mL, we examined PHI performance in the subcohort with levels of tPSA lower than 13 ng/mL and the AUC decreased to 0.694, but was still higher than tPSA (AUC = 0.382) and %fPSA (AUC = 0.630). 

However, the performance of PHI in identifying high-risk PCa from the non-aggressive PCa and BPHs is much higher than for identifying PCa from BPH in both the whole cohort and the subcohort, which can be explained because PHI correlates with the Gleason score, as has also been described previously by other studies [32]. 

The potential of % α2,3-SA to identify high-risk PCa has been confirmed in this study. The AUC was 0.97 with a cutoff of 30%, as previously described. Interestingly, % α2,3-SA performance was not influenced by the tPSA levels of the samples, and had the same performance in the subcohort of tPSA levels lower than 13 ng/mL.

% α2,3-SA test identifies PSA glycoforms containing α2,3-sialic acid, which have been linked to PCa aggressiveness [9,10,33]. PHI score comprises other PSA isoforms linked to PCa, namely [−2]proPSA, fPSA and tPSA. In this work, we have assessed whether these different PSA forms could complement each other to better identify high-risk PCa. The combination of both markers, % α2,3-SA and PHI, has given the best performance to identify high-risk PCa, with an AUC of 0.985 (100% sensitivity, 94% specificity), although larger independent cohorts are required to validate these promising results. In this regard, the methodology to determine the percentage of α2,3-sialic acid of PSA is currently being implemented to make it more automated so that it could be used in a clinical setting.

These results highlight that the future of prostate cancer diagnosis might rely on the combination of a panel of markers based on PSA forms that can give accurate molecular diagnosis and staging and indicate the likelihood of aggressive behavior.

## 4. Materials and Methods

### 4.1. Serum Samples

The study population included 79 patients (29 BPH and 50 PCa) from Hospital Universitari Dr. Josep Trueta (Girona, Spain) between 2006 and 2013. The study was approved by the Hospital Ethics Committee (Refs. 169.06 and 023.10) and all patients provided written informed consent before being enrolled. Patients’ sera were collected and stored at −80 °C. Urology and Pathology units from Hospital Universitari Dr. J. Trueta (Girona, Spain) performed the diagnosis using Transrectal Ultrasound-guided biopsy and/or adenomectomy/prostatectomy followed by pathological analysis.

The 29 BPH patients of the study (age range 44–76 years old) had a medical follow-up for a minimum of 2 years. 24 BPH patients had, at least, two negative biopsies with no evidence of high-grade Prostatic Intraepithelial Neoplasia (PIN). The 5 BPH left were subjected to prostate surgery (adenomectomy or prostate transurethral resection) and confirmed not to have prostate cancer by the Pathology Unit.

The 50 PCa patients of the study (age range 46–84 years old) were graded according to the Tumor-Node-Metastasis (TNM) classification following the general guidelines of the European Association of Urology. PCa patients were treatment naïve when serum samples were collected, except one PCa patient of the high-risk group, who was receiving hormonal therapy. High-risk PCa group comprised 22 patients with Gleason scores ≥8 (4 + 4) and/or with metastasis. The low-risk PCa group included 7 patients with Gleason scores of ≤6 (3 + 3), tPSA levels <10 ng/mL and clinical stage ≤pT2a. The group of intermediate-risk patients was comprised of 21 patients that did not meet the above criteria. They had Gleason scores of 7 (3 + 4 or 4 + 3) and 6 (3 + 3) and also included a patient with focal Gleason 8, tPSA levels <10 ng/mL and clinical stage pT2a considering his 10-year relapse-free survival. 

The average of tPSA serum levels for BPH patients was 7.59 ng/mL (range, 3.89 to 14.47 ng/mL). The average of tPSA for the PCa groups was: 17.83 ng/ml (range, 1.96 to 87.51 ng/mL) for high-risk PCa patients, 6.44 ng/mL (range, 3.73 to 12.42 ng/mL) for intermediate-risk PCa patients, and 4.56 ng/mL (range, 2.45 to 6.33 ng/mL) for low-risk PCa patients. 

### 4.2. Analysis of α2,3-Sialic Acid of Serum PSA

The determination of % α2,3-sialic acid of PSA was performed using a previously published method [9]. Briefly, ethanolamine 5 M was added to 0.75 mL of each serum sample to a final concentration of 1 M to release the PSA complexed to α1-antichymotrypsin. Total PSA was immunopurified using the Access Hybritech PSA assay Kit (Beckman Coulter, Brea, CA, USA). Amicon Ultra-0.5 3K Centrifugal Filter Devices (Millipore, Cork, Ireland) were used for desalting and concentrating the immunopurified tPSA samples up to a final volume of 40 μL. Samples were then applied to a lectin chromatography using *Sambucus nigra* (SNA)-agarose lectin (Vector Laboratories, Inc., Burlingame, CA, USA). Eluted unbound and bound chromatographic fractions were collected by centrifugation and quantification of free PSA of these fractions was performed using the Roche ELECSYS platform and used to determine the percentages of fPSA in the unbound fraction, corresponding to α2,3-sialic acid PSA, and in the bound fractions, which correspond to α2,6-sialic acid PSA.

### 4.3. Quantification of tPSA, fPSA and [−2]proPSA

Patient sera were analyzed for total PSA (tPSA), free PSA (fPSA), and [−2]proPSA on the Beckman Coulter Access 2 analyzer using WHO-standard-calibration. The Prostate Health Index (PHI) score was then calculated [PHI = ([−2]proPSA/fPSA) × $\sqrt{\mathrm{tPSA}}$]. Assays kits used were: Hybritech total PSA assay kit (Beckman Coulter, Fullerton, CA, USA; cat. no. 37200; Lot no. 523610), Hybritech free PSA assay kit (Beckman Coulter, Fullerton, CA, USA; cat. no. 37210; Lot no. 570228) and Hybritech p2PSA assay kit (Beckman Coulter, Fullerton, CA, USA; cat. no. P090026; Lot no. 527739). Assays were performed according to the instructions of their manufacturer and calibration and control materials used in each assay where the ones recommended by the manufacturer.

### 4.4. Statistics

Statistical analyses of both PHI and % α2,3-SA as PCa biomarkers were performed using IBM SPSS Statistics 23 for Windows and graphics were generated with SPSS software and GraphPad Prism 5 (GraphPad Software, Inc., La Jolla, CA, USA).

Patients were classified into four groups (BPH, low-risk PCa, intermediate-risk PCa, and high-risk PCa) and Shapiro-Wilk and Levene’s tests were used to assess the normality and homoscedasticity of variables. Differences of % α2,3-SA and PHI value between groups were analyzed using a Mann–Whitney *U* test. Receiver operating characteristic (ROC) curves were analyzed for tPSA, fPSA, % α2,3-SA, and PHI for distinguishing between high-risk PCa from the group of low-risk PCa, intermediate-risk PCa, and BPH, and also for distinguishing between PCa from BPH.

Bivariate regression (Pearson correlation) was used to analyze the correlation of % α2,3-SA and PHI with either the Gleason score or the tPSA levels. 

To combine PHI and % α2,3-SA, a logistic regression was performed, in which the response variable corresponded to the probability that the event of interest was a high-risk PCa (variable taking the value 1) or the group comprising low- and intermediate-risk PCa and BPH (variable taking the value 0). An R statistical package was used to develop a generalized lineal model (GLM) with binomial response. The construction and the comparison of the AUC of the ROC curves were performed using the Epi [34,35] and pROC libraries [36]. 

In all these analyses, *p* < 0.05 was considered statistically significant.

## Figures and Tables

**Table 1:** Clinical and pathological characteristics of the patients.

Pathology	Cases	*N*	PCa Recurrence, 1 Year	Gleason Score	*N*	Age Average	Range	tPSA ng/mL	±SD	Range	fPSA ng/mL	±SD	Range
**BPH**		29				63.24	44–76	7.59	2.39	3.89–14.47	1.26	0.53	0.30–2.28
**PCa *N* = 50**	Low-risk	7	0%	Gleason 5	1	84		2.45			0.27		
				Gleason 6	6	66.2	61–74	4.91	1.38	2.64–6.33	0.88	0.22	0.61–1.14
	Intermediate risk	21	4.8%	Gleason 6	5	56	47–75	5.79	3.73	3.73–12.42	0.53	0.33	0.19–0.97
				Gleason 7	15	65.2	46–78	6.61	1.84	5.13–10.39	0.65	0.29	0.58–1.36
				Gleason 8 focal	1	70		7.16			1.76		
	High-risk	22	59%	Gleason 7/metastasis	2	76	69–83	12.08	2.85	10.07–14.1	1.93	1.63	0.78–3.09
				Gleason 8	10	65.5	51–83	16.23	11.93	1.96–40.61	1.58	1.46	0.35–5.29
				Gleason 9	7	67.8	49–79	14.81	5.22	4.34–18.77	3.28	2.18	0.7–7.09
				Gleason 10	1	67		87.51			12.77		
				Gleason ND */metastasis	2	75	67–83	7.28	3.65	4.7–9.86	1.37	1.22	0.51–2.23

* ND: not determined; SD: Standard deviation.

## Abbreviations

PCa	Prostate cancer
PSA	Prostate Specific Antigen
PHI	Prostate Health Index
BPH	Benign Prostate Hyperplasia
AUC	Area Under the Curve
% α2,3-SA	Percentage of α2,3 sialic acid of PSA
DRE	Digital rectal examination
tPSA	Total PSA
fPSA	Free PSA
FDA	Food and Drug Administration
%fPSA	Free-to-total prostate-specific antigen ratio
PIN	Prostatic Intraepithelial Neoplasia
TNM	Tumor-Node-Metastasis
ACT	α1-Antichymotrypsin
SNA	*Sambucus nigra* lectin
CV	Coefficient of variation
mpMRI	Multi-parametric magnetic resonance imaging
